# Skeletal features in patient affected by maxillary canine impaction

**DOI:** 10.4317/medoral.18746

**Published:** 2013-05-31

**Authors:** Emanuele Mercuri, Michele Cassetta, Costanza Cavallini, Donatella Vicari, Rosalia Leonardi, Ersilia Barbato

**Affiliations:** 1 Research assistant, Department of Oral and Maxillo-Facial sciences, School of Dentistry, “Sapienza”,University of Rome, Rome, Italy; 2 Assistant professor, Department of Oral and Maxillo-Facial sciences, School of Dentistry, “Sapienza” , University of Rome, Rome, Italy; 3 Research assistant, Department of Radiology, “Sapienza”,University of Rome, Rome, Italy; 4 Professor, Department of Statistics, “Sapienza” University of Rome, Rome, Italy; 5 Professor, Department of Orthodontics, School of Dentistry, University of Catania, Catania, Italy; 6 Professor, Department of Oral and Maxillo-Facial sciences, School of Dentistry, “Sapienza” University of Rome, Rome, Italy

## Abstract

Objective: To analyze the skeletal features of patients with maxillary canine impaction. Material and Methods: The complete pre-treatment records of 1674 orthodontic patients were examined. From the subjects with maxillary impacted canine 12 patients were excluded , remaining 108. The subjects with maxillary impacted canine were divided into two study groups: a palatally displaced canine group (PDCG) (77 patients) and a buccally displaced canine group (BDCG) (31 patients). The values of the skeletal features measured on the lateral cephalometric radiograph were compared with a control group (CG) of 121 subjects randomly selected from the initial sample without maxillary canine impaction. The statistical analysis of the difference between the study groups and the CG was tested using ?2 test and Fisher’s exact test. The level of significance was set at P ?0.05. Results: The CG was characterized by increased values of A point-Nasion-B point angle (ANB) and by a retro-positioned or smaller lower jaw. PDCG patients showed normal skeletal features compared to the CG, presenting mainly I class and lower rank of II and III sagittal skeletal features. PDCG subjects presented also normal values of the Steiner vertical skeletal relationship angles with normal facial divergence compared to the CG. PDCG cases were also characterized by horizontal and prognathic growth. BDCG did not present significant differences in skeletal features compared to the CG, except for an increased ANB. Conclusions: Palatally displaced canine (PDC) was frequently the only orthodontic problem of patients and was not associated whit altered skeletal features. The frequent absence of malocclusion in PDC patients explains the delayed identification of this problem. BDCG patients did not present significant differences in skeletal features with respect to the orthodontic population. The presence of both buccally displaced canine (BDC) and malocclusion makes the patient with BDC both aware of the need for, and motivated to undergo, orthodontic treatment.

** Key words:**Canine impaction, palatal displacement, buccal displacement, skeletal features.

## Introduction

After the third upper molar, the maxillary canine is the most common tooth that can undergo impaction, with a prevalence of 1-3% (1-6). In spite of the large number of publications on this subject, the aetiology of maxillary canine impaction is still under discussion. While numerous factors determining impaction are being assessed, it is certain that a buccally displaced canine (BDC) and a palatally displaced canine (PDC) are characterized by different ethiopatogenesis (7). BDC is thought to be a form of crowding: insufficient space in upper arch for the eruption of the maxillary canine culminates in its impaction; nevertheless, given time and space this tooth will usually erupt in the oral cavity (8). On the other hand, PDC often occurs in patients who do not present crowded arches or even an excess of space in the canine area (7-9). The aetiology of PDC is still unclear. Some authors believe that the absence of lateral incisor guidance (guidance theory) could lead to palatal canine impaction by allowing the canine to cross back from the buccal to the palatal side (10-13). Indeed, a link between PDC and upper lateral incisor anomalies has been demonstrated, as has an association with smaller mesio-distal crown width and shorter roots of the maxillary lateral incisor (10). In spite of these considerations, a great number of studies have put forward a “genetic theory” (7) of PDC: thanks to the assessment of the simultaneous occurrence of PDC and congenital dental anomalies (peg shaped lateral incisor, aplasia or impaction of other teeth) the authors of these studies believe that PDC is only one of the aspects of a general dental eruption disorder which could be genetic in origin (14-24). Few studies report research about the skeletal features of patients with maxillary impacted canines (24-27).

The aim of this study is to determine the main skeletal features in subjects with buccally (BDC) and palatally (PDC) displaced canines.

## Material and Methods

In this study an analysis of the pretreatment records of 1674 Caucasian patients treated at Department of Orthodontics of “Sapienza” University of Rome and at the Department of Orthodontics of the University of Catania, Italy, was performed. This study followed the Declaration of Helsinki on medical protocol and ethics, and the regional Ethical Review Board of the ‘‘Umberto I’’ General Hospital of Rome approved the study. Caucasian patients with at least one impaction of a maxillary canine were selected for the study. The impaction diagnosis and the impaction site were determined on the basis of clinical examinations and standardized radiographs (panoramic x-ray, computed tomography, intraoral radiographs). Subjects with a cleft lip or palate or other craniofacial syndromes associated with tooth aplasia or displacement, trauma and multi-reagent chemotherapy were excluded. From the entire study population with maxillary canine impaction, two study groups were created: palatally displaced canine group (PDCG) and buccally displaced canine group (BDCG). From the study groups 12 patients were excluded because the pre-treatment lateral cephalometric radiographs were missing, leaving 77 subjects in the PDCG and 31 in the BDCG. They were compared with a control group (CG) of 121 subjects randomly selected from the study population without maxillary canine impaction. In order to determine the association of PDC and BDC with cephalometric features, the three different groups (PDCG, BDCG, CG) were subsequently compared as reported below:

1 PDCG vs. CG

2 BDCG vs. CG

Cephalometric measurements were performed using a lateral cephalometric radiograph. The tracings were created by the same author (E.M.), made on ultrathin 0.003 inch transparent acetate sheets using a Pentel 0.3 mm lead pencil. All the cephalometric radiographs were evaluated on a masked, illuminated viewbox in a room with reduced lighting and were measured manually. The cephalometric parameters evaluated in this study to estimate the craniofacial skeletal relationship were ( Table 1):

**Table 1:**
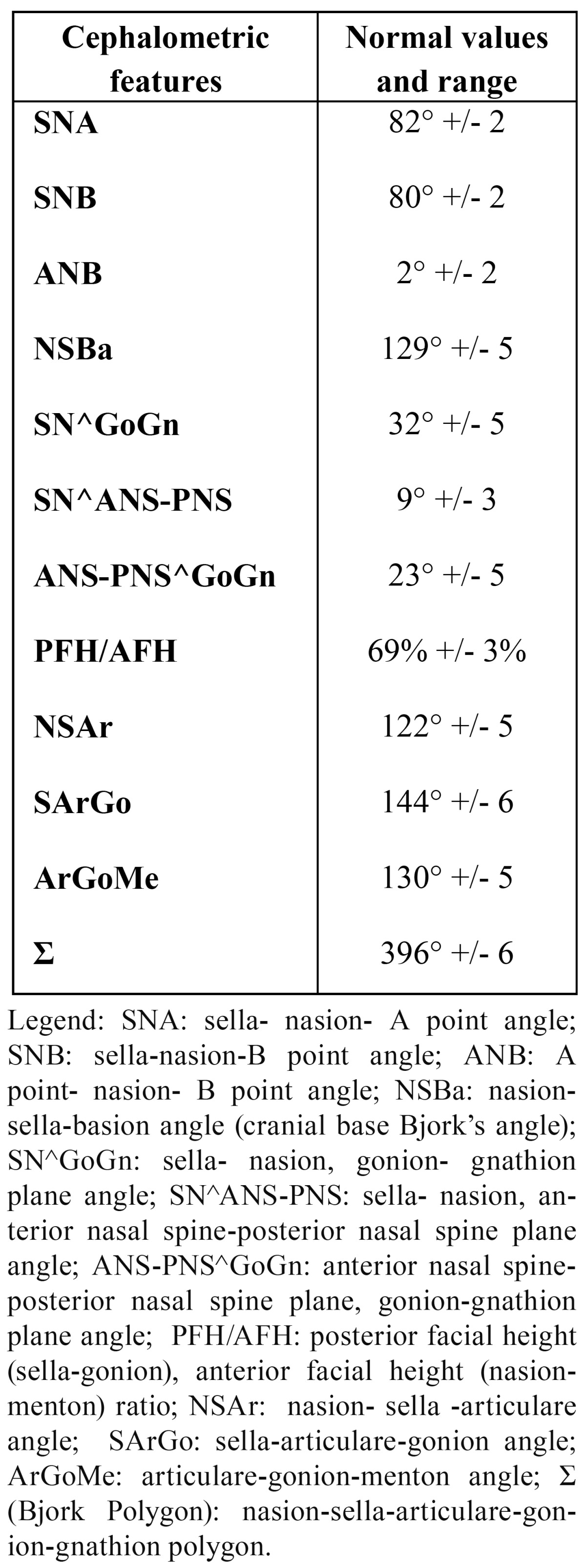
Normal values and range of cephalometric features.

- sagittal parameters: sella- nasion- A point angle (SNA), sella-nasion-B point angle (SNB), A point- nasion- B point angle (ANB), 

- cranial base Bjork’s angle (NSBa),

-vertical parameters: sella-nasion, gonion-gnathion plane angle (SN^GoGn); sella-nasion, anterior nasal spine-posterior nasal spine plane angle (SN^ANS-PNS); anterior nasal spine-posterior nasal spine plane, gonion-gnathion plane angle (ANS-PNS^GoGn) and posterior facial height(sella-gonion), anterior facial height (nasion-menton) ratio (PFH/AFH) of Jaraback, to evaluate the craniofacial skeletal divergence;

- growth parameters of Jarabak: nasion-sella -articulare angle (NSAr), sella-articulare-gonion angle (SArGo), articula-re-gonion-menton angle (ArGoMe), nasion-sella-articulare-gonion-gnathion polygon (?).

All the parameters measured were classified into three categories (normal, increased and decreased). The normal values and range of cephalometric features were described in the  Table 1. The reproducibility of cephalometric measurements was assessed by re-examining the lateral cephalometric radiographs of 25 patients 2 weeks after the first examination by a single operator. Reproducibility was 100% for all variables except for NSBa angle (98%). Statistical analysis was performed at the Department of Statistics (“Sapienza” University of Rome) using SAS® software (Statistical Analysys System, IBM SAS Institute inc). The prevalence and the distribution were evaluated for each variable. The analysis of significant associations was performed using the ?2 test, which was assumed to be significant when the p-value was not greater than 0.05 (P ?0.05). When the counts of some variables were small, given that the ?2 test may be not appropriate, Fisher’s exact test was used. In order to evaluate the association of each category of statistically significant cephalometric features, the subsamples (normal, increased and decreased) were evaluated using the ?2 test again. Taking the significant variables into consideration, the odds ratios were evaluated as an approximation of the relative risk. The higher the value of the odds ratio, the higher the probability of canine impaction occurring in the presence of the relevant skeletal anomaly.

## Results

The BDCG consisted of 22 (70.97%) females and 9 (29.03%) males (sex ratio 2:1), aged between 10 and 22 years old (mean age: 13.64; SD 3.39). The PDCG was composed of 50 (64.94%) females and 27 (35.06%) males (sex ratio 2:1), aged between 11 and 40 years old (mean age: 14.32; SD 5.33). The CG consisted of 121 subjects: 65 (53.72%) females and 56 (46.28%) males (sex ratio:1:1), aged between 10 and 40 years old (mean age: 11.89; SD 4.361). In  Table 2 the distributions for all the cephalometric parameters investigated are reported for the three groups of subjects ( PDCG, BDCG, CG ). The ?2 test and Fisher’s exact test p-values are shown in Table 3.  Table 3 displays statistically significant associations (p-value?0.05) between PDC and SNB, ANB, SN^GoGN, SN^ANS-PNS, ANS-PNS^GoGn, PFH/AFH, ?, while no significant associations emerged between PDC and SNA, SNBa and the growth parameters of Jarabak, except for ?. In order to evaluate in which direction such associations are significant, for each significant skeletal feature the association of the abnormal (increased or decreased) feature with the presence of canine impaction was tested again , in the appropriate subsamples of patients, by the ?2 test.  Table 4 shows that in comparing PDCG vs CG, SNB and ? results significantly decreased with respect to the normal value, while for ANB, SN^GoGN, SN^ANS-PNS, ANS-PNS^GoGn, PFH/AFH significant associations with the palatal canine impaction resulted for the increased features.  Table 4 also shows the odds ratios and the corresponding confidence intervals for each significant comparison. No significant associations were found between BDC and the skeletal features, except for ANB angle ( Table 3) and, specifically, for the increased ANB ( Table 4).

**Table 2:**
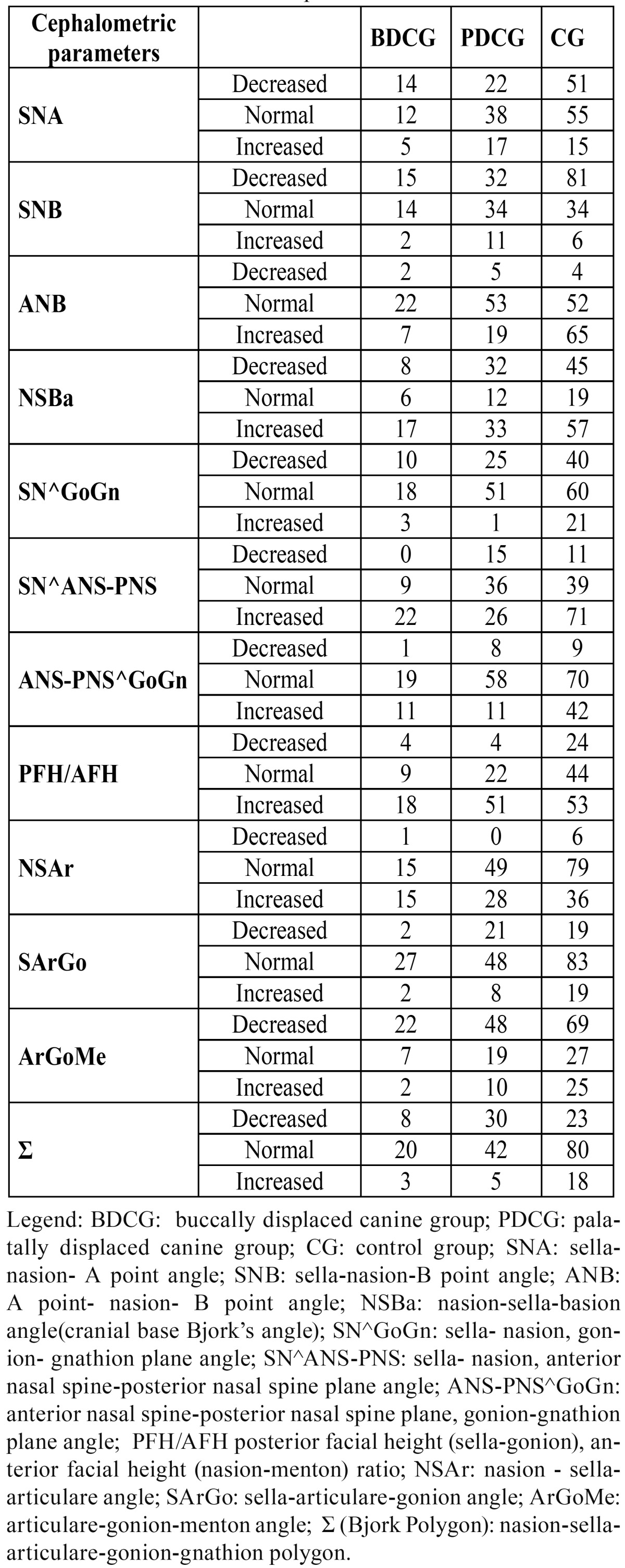
Distributions of the cephalometric features.

**Table 3:**
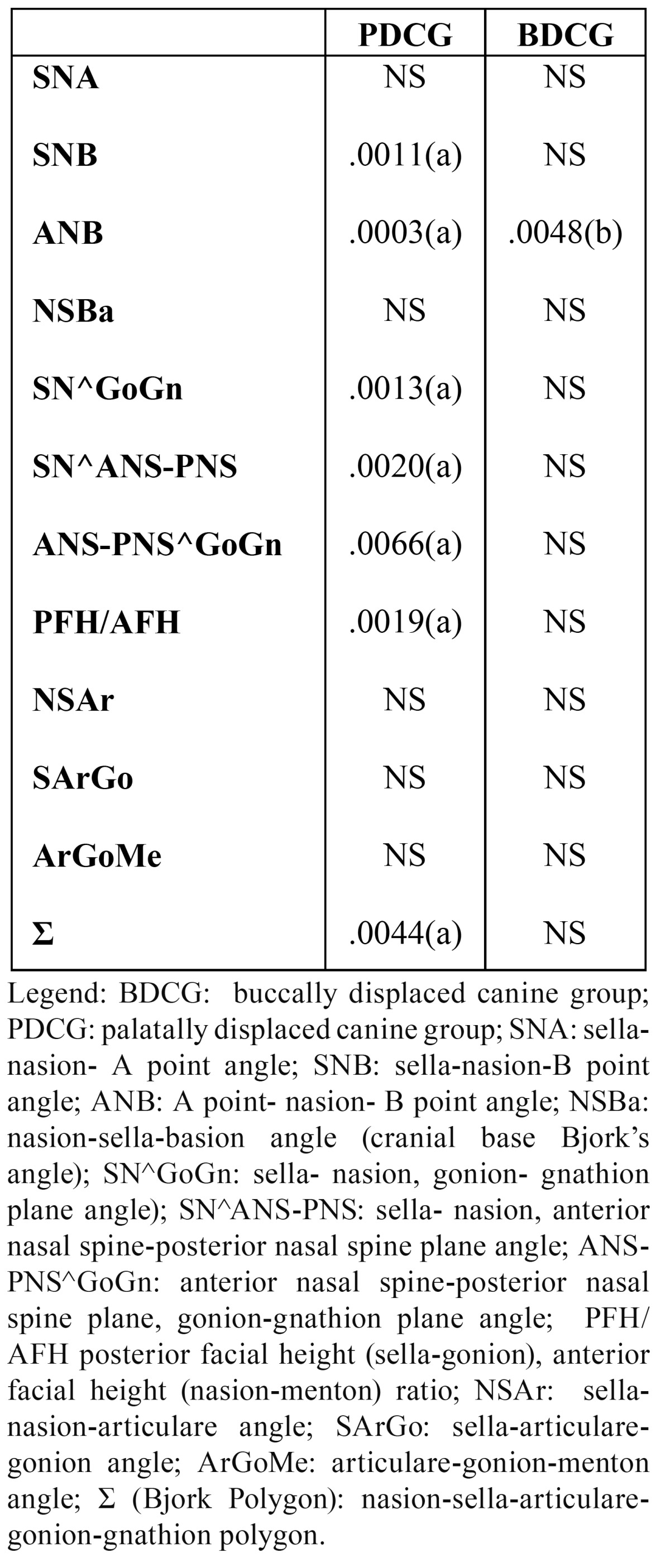
P-values for cephalometric features. NS: not significant; (a): ?2 test p-value; (b): Fisher's exact test p-value. Significance was set to P ?0.05.

**Table 4:**
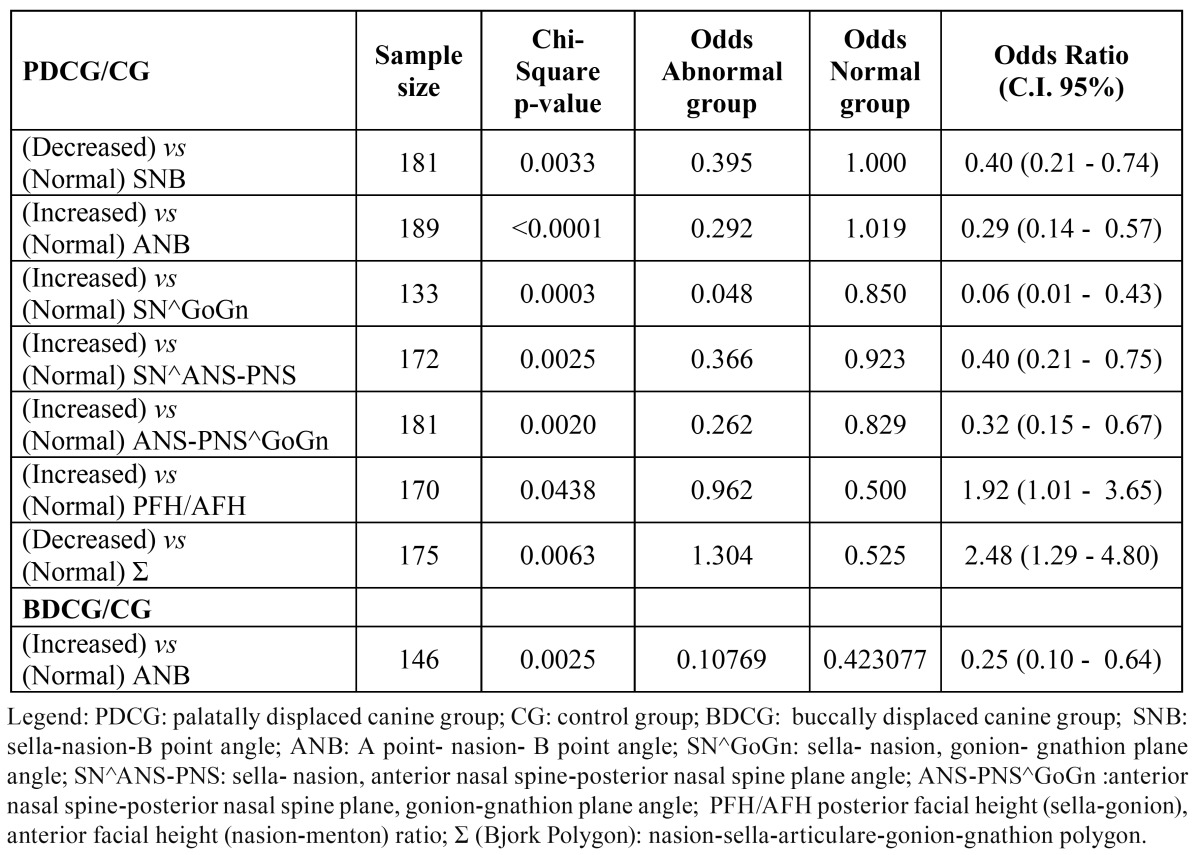
Odds for Cephalometric features: PDCG/CG and BDCG/CG.

## Discussion

In order to assess the data in this study, the fact that the CG was composed of orthodontic patients with an high percentage of malocclusion must be taken into consideration. The results must be regarded critically with the CG profile in mind, carefully evaluating the distributions of each feature in the groups. In particular, it must be pointed out the high frequency of abnormal cephalometric variables in the CG. Moreover, it must be highlighted the relative scarcity of studies related to the cephalometric features of impacted maxillary canines in the literature. The analysis of skeletal features resulted in statistically significant associations between PDC and normal values of SNB and ANB (Class I). Whereas no statistical association was found for SNA and SNBa angles. The Angle Class II is the most frequent sagittal malocclusion that affected the Caucasian population, so the CG was characterized by increased values of ANB angle and this is often caused by a retropositioned or smaller lower jaw. On the contrary, PDC patients usually presented a high percentage of normal cephalometric variables: showing a high prevalence of Class I craniofacial skeletal relationships. In fact, PDC was frequently the only orthodontic problem of the patient. The absence of malocclusion in PDC patients plays an important role in its diagnosis and prognosis, indeed it could explain the delayed identification of this problem, thwarting the use of preventive therapies (28). The coexistence of a malocclusion is usually the reason why PDC patients request an orthodontic examination and treatment: these patients are often not aware of their problem and even when it is diagnosed, they are not inclined to resolve it. In order to overcome any complacency with respect to the state of their dental health and thereby motivate them to take action, the patient needs to be fully-informed of the implications of PDC (28). The craniofacial skeletal relationship in subjects with canine impaction has been studied previously, with no clear association to any specific craniofacial pattern (24-27). Sacerdoti et al. (24), in evaluating the ANB angle in PDC patients, determined that the sagittal skeletal relationship was very similar to the standard orthodontic population but this data has not been confirmed by the present study. Basdra et al. (25), associated canine impaction with Class II Division 2 malocclusions, showing a 33.5% of canine impaction in subjects with this type of malocclusion. In 2001 Basdra et al. (26) affirmed that 9% of Class III subjects and 1.33% of Class II Division 1 subjects were affected by canine impaction. In the current study PDCG frequently presented normal SN^GoGn, SN^ANS-PNS, ANS-PNS^GoGn values in comparison with the CG. No statistical association was found between BDCG and vertical skeletal parameters. Few studies have reported an association between the vertical skeletal relationship and PDC; the results of these studies have shown an association between PDC and occlusal deep bite (24,27). Sacerdoti et al. (24) , reported a three times higher prevalence rate for hypodivergent cases in the PDC subjects compared to the control cases. With regard to PFH/AFH , the present study revealed an association with PDCG, moreover a higher risk of PDC was found particularly when the subject presents increased values of this parameter or in cases of horizontal growth; no statistical association was found between PDCG and CG for each individual growth angle of Jarabak ( NSAr, SArGo, ArGoMe ). PDCG also presented statistically significant results for ? growth parameter of Jarabak compared to the CG: in particular, resulted in a higher risk for decreased values, therefore for the prognathic growth.

The results of this study show that subjects with PDC have mostly normal skeletal features, showing Class I skeletal relationships and lower ranks of II and III sagittal skeletal features in comparison to the orthodontic population. Moreover PDC patients present normal values of the Steiner vertical skeletal relationship angles, or rather they are more likely for a normal facial divergence compared to the orthodontic population studied. PDC is also characterized by horizontal and prognathic growth. The frequent absence of malocclusion in PDC patients explains the delayed identification of this problem, which does not allow for the use of preventive therapies. These patients in fact are generally not otherwise conscious of their problem and sometimes they lack of motivation. Conversely, the BDC group did not present significant differences in the skeletal features compared to the orthodontic population. The presence of both BDC and malocclusion makes the patient both aware of the need for, and motivated to undergo, orthodontic treatment.
